# P-692. Early host blood transcriptional signatures predictive of lower respiratory tract disease progression during parainfluenza infection in hematopoietic cell transplant (HCT) recipients

**DOI:** 10.1093/ofid/ofae631.888

**Published:** 2025-01-29

**Authors:** Fang Yun Lim, Anna Nordlander, Chikara Ogimi, Linda M Sircy, Terry L Stevens-Ayers, Ashleigh Theberge, Sina A Gharib, Michael J Boeckh, Alpana waghmare

**Affiliations:** Fred Hutchinson Cancer Center, Seattle, Washington; Karolinska University Hospital, Stockholm, Stockholms Lan, Sweden; National Center for Child Health and Development, Setagaya-ku, Tokyo, Japan; Fred Hutchinson Cancer Center, Seattle, Washington; Fred Hutchinson Cancer Center, Seattle, Washington; University of Washington, Seattle, Washington; University of Washington, Seattle, Washington; Fred Hutchinson Cancer Center, Seattle, Washington; Fred Hutchinson Cancer Center; Seattle Children's Hospital, Seattle, Washington

## Abstract

**Background:**

Parainfluenza virus (PIV) lower respiratory tract infection (LRTI) following HCT is associated with increased mortality and post-transplant complications. Clinical risk scores for progression from upper respiratory tract infection (URTI) to LRTI have limited predictive value and host transcriptional profiles may improve risk stratification.

**Figure 1.**
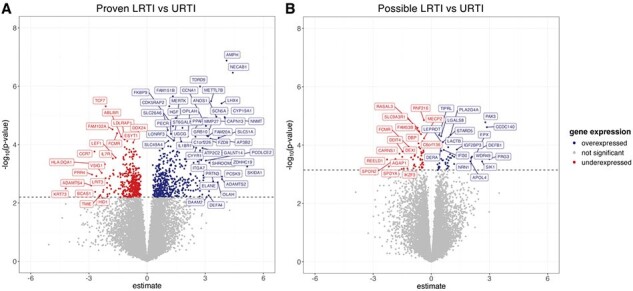
URTI samples from proven LRTI progressors showed robust differences in transcriptional signatures compared to URTI samples from non-progressors.

Significant genes identified from comparisons of URTI samples from A) proven LRTI progressors vs non-progressors and B) possible LRTI vs non-progressors. Volcano plots depict significantly overexpressed (blue) and underexpressed (red) genes. Y-axis denotes the p-values while x-axis denotes estimates for each comparison. Horizontal dashed line denotes an adjusted p-value cut off < 0.1.

**Methods:**

All blood samples were collected in HCT recipients at the time of PIV URTI. Subjects were followed for progression to LRTI and classified as: URTI (non-progressors), possible LRTI progressors (upper tract PIV detection with radiographic changes), or proven LRTI progressors (lower tract PIV detection with radiographic changes). Libraries were prepared using TruSeq mRNA Stranded Library Kit and sequenced on the NovaSeq 6000. Linear models of gene expression and comparisons for “possible LRTI vs URTI” and “proven LRTI vs URTI“ were fit using kimma. An adjusted p-value < 0.1 (Benjamini-Hochberg) defined significant genes. Gene ontology enrichment analyses of overexpressed and underexpressed genes were performed using clusterProfiler.

**Figure 2.**
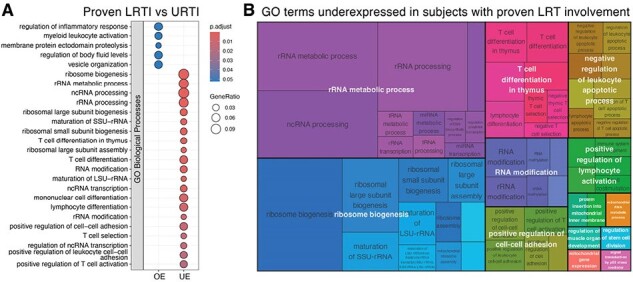
Gene ontology (GO) enrichment analyses of overexpressed (OE) and underexpressed (UE) genes in URTI samples from proven LRTI progressors. A) Dotplot of top GO terms within the Biological Processes (BP) sub-ontology enriched in each OE and UE gene cluster from proven-LRTI vs URTI comparisons. B) Treemaps of reduced GO terms underexpressed in URTI samples from proven LRTI progressors. Terms are grouped (colored) based on their reduced parent terms (white fonts) and the space used by the term is proportional to the enrichment score.

**Results:**

46 subjects with PIV infection following HCT (2010 - 2019) were included (URTI = 32; possible LRTI = 7; proven LRTI = 7). Robust differences in gene expression (881 genes, Fig. 1A) were observed in URTI samples from proven progressors relative to non-progressors, while possible LRTI progressors showed higher similarity to non-progressors (102 genes, Fig. 1B). Notably, processes involved in rRNA metabolism, ribosome biogenesis, and T cell differentiation were underexpressed in proven LRTI progressors, whereas immunoinflammatory pathways were overexpressed (Fig. 2A and 2B).

**Conclusion:**

We demonstrated that early PIV URTI blood samples can identify predictive signatures of progression to LRTI in HCT recipients presenting with URTI. Underexpression of T cell immunity and ribosome/rRNA metabolic pathways in proven progressors suggest a protective role in reducing risk of progression to PIV LRTI in HCT recipients. Early stratification of patients at risk of progression using early host blood transcriptional signatures may be useful to guide treatment management, reducing overall mortality rate and disease burden in HCT recipients.

**Disclosures:**

**Chikara Ogimi, MD, PhD**, AstraZeneca: Honoraria|bioMerieux Japan Ltd.: Honoraria|ELSEVIER: Honoraria|KYORIN: Honoraria|Miyarisan: Honoraria|MSD: Honoraria|NOVARTIS: Honoraria|Pfizer: Honoraria **Michael J. Boeckh, MD PhD**, Allovir: Advisor/Consultant|Allovir: Grant/Research Support|AstraZeneca: Advisor/Consultant|AstraZeneca: Grant/Research Support|Merck: Advisor/Consultant|Merck: Grant/Research Support|Moderna: Advisor/Consultant|Moderna: Grant/Research Support|Symbio: Advisor/Consultant **Alpana waghmare, MD**, Allovir: Grant/Research Support|Ansun Biopharma: Grant/Research Support|GlaxoKlineSmith: Advisor/Consultant|GlaxoKlineSmith: Grant/Research Support|Pfizer: Grant/Research Support|Vir: Advisor/Consultant

